# Moxibustion for the Correction of Nonvertex Presentation: A Systematic Review and Meta-Analysis of Randomized Controlled Trials

**DOI:** 10.1155/2013/241027

**Published:** 2013-09-15

**Authors:** Qin-hong Zhang, Jin-huan Yue, Ming Liu, Zhong-ren Sun, Qi Sun, Chao Han, Di Wang

**Affiliations:** ^1^Department of Acupuncture and Moxibustion, College of Acupuncture and Moxibustion, Heilongjiang University of Chinese Medicine, Harbin 150040, China; ^2^The Second Affiliated Hospital, Heilongjiang University of Chinese Medicine, Harbin 150040, China; ^3^College of Basic Medical Sciences, Heilongjiang University of Chinese Medicine, Harbin 150040, China

## Abstract

*Objectives*. This study aims to assess the effectiveness and safety of moxibustion for the correction of nonvertex presentation. *Methods*. Records without language restrictions were searched up to February 2013 for randomized controlled trials (RCTs) comparing moxibustion with other therapies in women with a singleton nonvertex presentation. Cochrane risk of bias criteria were used to assess the methodological quality of the trials. *Results*. Seven of 392 potentially relevant studies met the inclusion criteria. When moxibustion was compared with other interventions, a meta-analysis revealed a significant difference in favor of moxibustion on the correction of nonvertex presentation at delivery (risk ratio (RR) 1.29, 95% confidence interval (CI) 1.12 to 1.49, and *I* ^2^ = 0). The same findings applied to the cephalic presentation after cessation of treatment (RR 1.36, 95% CI 1.08 to 1.71, and *I* ^2^ = 80%). A subgroup analysis that excluded two trials with a high risk of bias also indicated favorable effects (RR 1.63, 95% CI 1.42 to 1.86, and *I* ^2^ = 0%). With respect to safety, moxibustion resulted in decreased use of oxytocin. *Conclusion*. Our systematic review and meta-analysis suggested that moxibustion may be an effective treatment for the correction of nonvertex presentation. Moreover, moxibustion might reduce the need for oxytocin.

## 1. Introduction 

Moxibustion is a traditional Chinese medical intervention that utilizes the heat generated by burning herbal preparations containing Artemisia vulgaris (mugwort) to stimulate acupuncture points [1]. It is also believed to be effective in the treatment of stroke rehabilitation [2], pain [3], cancer care [4], ulcerative colitis [5], hypertension [6], osteoarthritis [7], constipation [8], child chronic cough [9], and breech presentation [10]. In China, moxibustion on the *Zhiyin* (BL67) point, located on the outer corner of the fifth toenail, has long been used to correct nonvertex presentation in obstetrics [11, 12]. Possible mechanisms of action attributed to moxibustion include stimulation of the production of placental oestrogens, alterations in prostaglandin levels, and promotion of the uterine contractility, which leads to a stimulation of fetal movements and a higher probability of vertex presentation of the fetus [10, 12–14].

Before moxibustion can be recommended for routine clinical use for the correction of non-vertex presentation, evidence from randomized controlled trials is required. Unfortunately, most studies in which the moxibustion has been evaluated are open clinical trials, blinded to neither the practitioner nor the subjects. In moxibustion trials, sham treatments are conducted by adding insulation below the moxa pillar to prevent the transfer of heat from the pillar to the patient [15]. The sham treatment looks similar to the real moxibustion treatment in appearance and burning procedure, and participants are able to smell the smoke or observe the burning moxa [15].

The efficacy of moxibustion for the correction non-vertex presentation has been evaluated in four clinical reviews [23–26]. All four studies failed to include all of the relevant articles published [23–26]. For example, none of these reviews included the study of Yang and colleagues [16], which met all of the inclusion criteria for each of the four reviews. Additionally, all of these reviews included interventions other than moxibustion including acupuncture [23–26]. Finally, some reviews included controlled clinical trials [23] and quasirandomised controlled trials [24–26] which were poorly executed and might have affected the conclusion of the reviews. 

The objective of the current review and meta-analysis was to perform a comprehensive literature search to find and evaluate high-quality RCTs. Also, our study aim was to critically evaluate the clinical efficacy and safety of moxibustion therapy alone for the correction of non-vertex presentation (not combined with acupuncture or acupuncture alone).

## 2. Materials and Methods

### 2.1. Literature Search

The comprehensive literature search included the following electronic databases: MEDLINE (1950 to February 2013), EMBASE (1980 to February 2013), Cochrane Library (1980 to February 2013), CINAHL (1982 to February 2013), AMED (1985 to February 2013), British Nursing Index (1993 to February 2013), Chinese Biomedical Literature Database (CBM; 1980 to February 2013), China National Knowledge Infrastructure (which includes the database China Academic Journals) (CNKI; 1980 to February 2013), VIP Information (VIP; 1980 to February 2013), Wanfang Data (WAN FANG; 1980 to February 2013), Science paper Online (2006 to February 2013), and 28 major Chinese traditional medicine journals. 

The following search terms were used: moxibustion OR moxa AND non-vertex presentation or labor presentation or abnormal foetal position or abnormal foetal presentation or podalic presentation or complementary medicine or alternative medicine. We also performed a hand search to identify any other articles. In an attempt to minimize the omission of potentially relevant trials, we also reviewed the reference lists of included articles and relevant reviews for additional eligible studies. Both published and unpublished studies were considered. No language restrictions were imposed.

### 2.2. Selection of Studies

Potentially relevant studies were independently evaluated by two reviewers (Y. J. H. and Z. Q. H.). Reviewers screened all titles and abstracts when available and they examined the full text if the study met the following inclusion criteria: (a) was a RCT; (b) included a comparison of moxibustion with nonmoxibustion therapy; and (c) included no restriction on the race or gestation of participants with a singleton non-vertex presentation. However, the study with following criteria was excluded: (a) duplication; (b) complex therapy that could not figure out the effect of moxibustion for example, treatment group used moxibustion plus Chinese herbal ointment, while the control group used knee-chest therapy; (c) incomplete data (failed to provide basic characteristics of participants, such as age, gestational week, and duration of intervention); and (d) wrong intervention or comparator that could not evaluate the effect of moxibustion; for example, treatment group used moxibustion plus acupuncture intervention, while control group used moxibustion intervention. Disagreements between the two reviewers were resolved by discussion with a third author (S. Z. R.) to achieve consensus. 

### 2.3. Outcome Measures

In this review, we present the results for the cephalic presentation at birth and after cessation of treatment. In addition, use of oxytocin, Apgar scores less than 7 at 5 minutes, cesarean section, preterm delivery, premature rupture of membranes, intrauterine fetal death, placental abruption, and cord blood pH less than 7.1 were also recorded.

### 2.4. Data Extraction

Two authors (S. Q. and H. C.) independently extracted data from eligible studies using a predesigned extraction sheet and a third author (W. D.) verified the extracted data. Any discrepancies were settled through discussion. The third review author (W. D.) was consulted if a consensus could not be reached. The extracted data included demographic data, clinical characteristics of the study groups, quality of trial design, inclusion and exclusion criteria, interventions, results, and adverse events. If the required information was not available in the included studies, we contacted the original authors by email. 

### 2.5. Quality of the Studies

The Cochrane risk of bias tool [27] was used to assess methodological quality of the trials. Two authors (Y. J. H. and Z. Q. H.) were independently involved in quality assessment. All discrepancies were resolved by consensus with the other author (L. M.).

### 2.6. Statistical Analysis

Data were pooled using the random-effects model. Treatment effect was expressed as a relative risk, and 95% confidence intervals (CIs) were calculated. Heterogeneity was evaluated using Cochrane's Tau², *I* ^2^, and Chi² statistics, and high heterogeneity was assumed if the Tau² was greater than zero and either the *I* ^2^ was greater than 30% or *P* value was less than 0.10 in the Chi² test [27]. Subgroup analysis was conducted to identify and explain heterogeneity. Where possible, a funnel plot was used to assess publication bias. We also performed post hoc sensitivity analysis to test the robustness of the overall effect.

## 3. Results

### 3.1. Study Description

**Figure 1:**
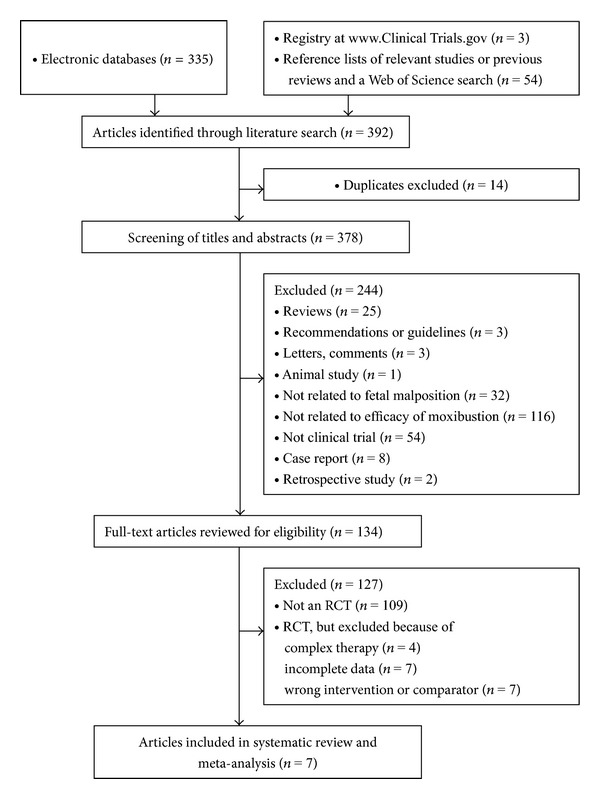
Flowchart of study selection.

We identified 392 potentially relevant articles. Seven RCTs, including a total of 1387 participants, met our inclusion criteria [16–22] (Figure 1). The characteristics of the 7 trials are summarized in Tables 1 and 2. Of those 7 RCTs, four studies were from Western countries and published in English [18–21], while the other three trials were from China [16, 17, 22], one published in English [17] and two in Chinese [16, 22].

Four trials compared moxibustion therapy with observation [17, 18] and usual care [19, 20]. Two studies compared moxibustion therapy with postural techniques [16, 21], and one study compared moxibustion plus postural technique therapy with postural measures [22].

### 3.2. Study Quality

**Figure 2:**
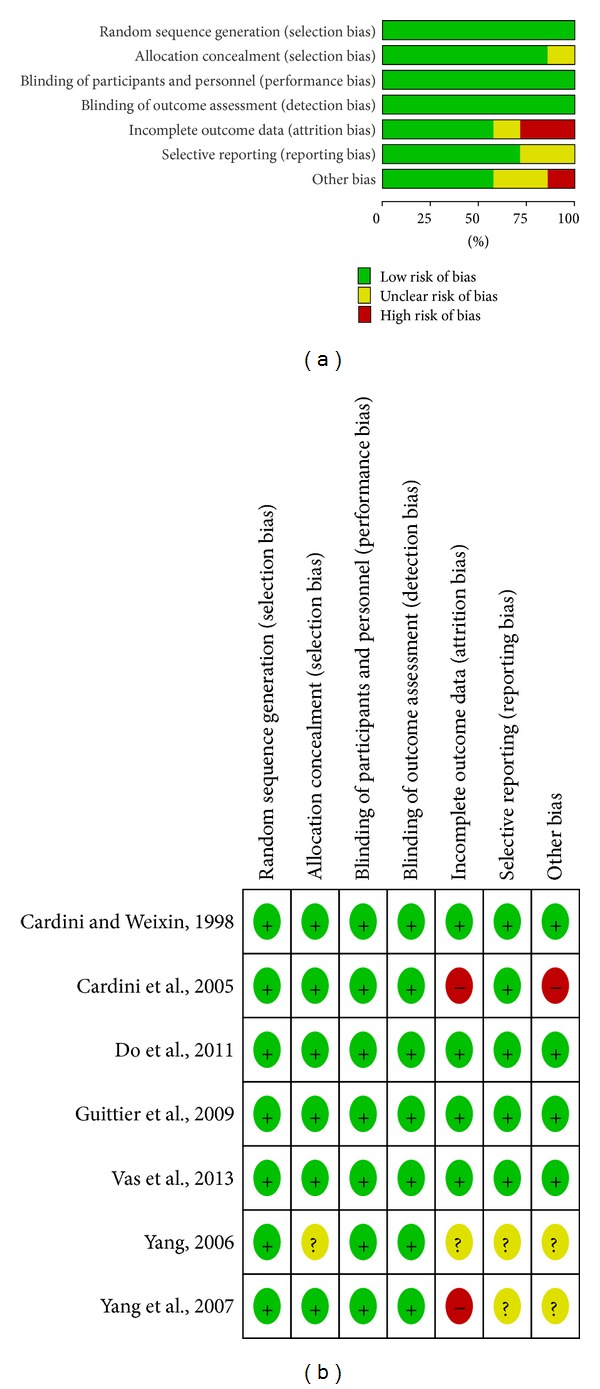
(a) Risk of bias graph: review authors' judgments about each risk of bias item presented as percentages across all included studies. (b) Risk of bias summary: review authors' judgements about each risk of bias item for each included study.

The Cochrane risk of bias was presented in Figures 2(a) and 2(b) and Table 3. All seven RCTs reported appropriate sequence generation [16–22]. Six studies conducted concealment of allocation by sealed envelopes [16–21], while one trial did report it [22]. In five studies, moxibustion was either applied at home by participants themselves [17–19, 21] or by practitioners in hospital [16, 20], while the remaining one study did not state who applied the intervention [22]. In that study, it was not feasible to blind the participant or the therapist. Although the outcome assessor was blinded in only one study [18] and the analyst was blinded to groups in three studies [16, 19, 21], the review authors deemed that the outcomes and their measurements were not likely to be influenced by lack of blinding. Thus, all studies had a low risk of bias with the Cochrane risk of bias tool at blinding levels. Four studies reported complete followup of all subjects [17–21]. One study stated that 7 women from treatment group and 10 women from control group withdrew from the trial [16]. One trial reported that 1 woman was lost to followup in the control group, and 14 women discontinued treatment in the intervention group [18]. The other one did not provide any information of followup [22]. When it comes to selective reporting bias, the trial protocol was available for two trials [19, 21]; however, the other five studies failed to provide it [16–18, 20, 22]. Of those five trans, three studies included all expected outcomes [17, 18, 20], while the remaining two failed to state them, so the review authors were unable to determine whether all outcomes were prespecified [16, 22]. All seven trials conducted sample size calculations [16–21], except for one study that did not report it [22]. Five trans did not report imbalances at randomization, and they appeared free of other sources of bias [17, 19–22]. One study failed to provide sufficient information, so the review author did not determine whether the other bias is present [16]. The other one was interrupted when interim analysis revealed poor compliance and a high number of treatment interruptions [18]. 

### 3.3. Outcome Measures

**Figure 3:**
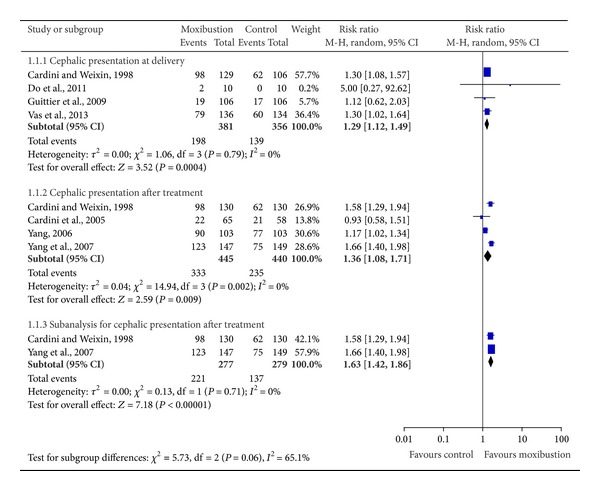
Effectiveness of moxibustion for the correction of non-vertex presentation.

**Figure 4:**
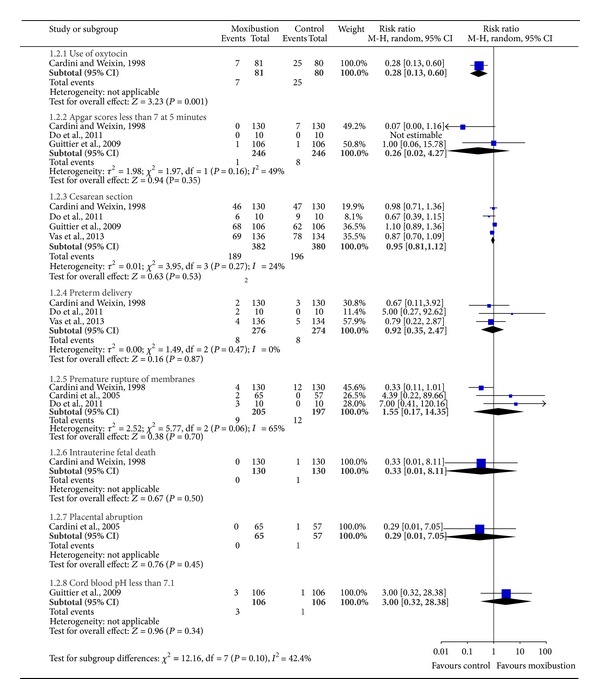
Safety of moxibustion for the correction of non-vertex presentation.

Seven included trials assessed the effect of moxibustion (alone or in association with postural techniques) compared with observation alone or postural measures on cephalic presentation at delivery [17, 19–21] and after cessation of treatment [16–18, 22] (Figure 3). Five out of the seven studies involved the other outcomes of safety on the use of oxytocin [17], Apgar scores less than 7 at 5 minutes [17, 19, 20], cesarean section [17, 19–21], preterm delivery [17, 19, 21], premature rupture of membranes [17–19], intrauterine fetal death [17], placental abruption [18], and cord blood pH less than 7.1 [20] (Figure 4).

 Our meta-analysis of four studies [17, 19–21], which included 737 participants, yielded encouraging effects in favor of moxibustion on cephalic presentation at delivery (excluding ECV) (RR 1.29, 95% CI 1.12 to 1.49, and *I* ^2^ = 0) (Figure 3). The same findings applied to the cephalic presentation after cessation of treatment, when moxibustion (alone or in combination with postural techniques) was compared with observation [17, 18] or postural techniques [16, 22] (RR 1.36, 95% CI 1.08 to 1.71, and *I* ^2^ = 80%) (Figure 3). A subgroup analysis that excluded two studies with a high risk of bias [18, 22] showed significant effect of moxibustion (RR 1.63, 95% CI 1.42 to 1.86, and *I* ^2^ = 0%) (Figure 3). 

Five trials examined the safety of moxibustion for the correction of non-vertex presentation [17–21] (Figure 4). One study reported significant differences in favor of a reduced use of oxytocin in the treatment group [17] (RR 0.28, 95% CI 0.13 to 0.60) (Figure 4). No other statistically significant differences were found in the comparison between moxibustion treatment group and nomoxibustion group on Apgar scores less than 7 at 5 minutes, cesarean section, preterm delivery, premature rupture of membranes, intrauterine fetal death, placental abruption, and cord blood pH less than 7.1 (Figure 4). 

### 3.4. Adverse Events

Three trials reported the adverse events in the moxibustion group [17–19]: two reported two and four cases of premature deliveries at 37 weeks, respectively. Four cases of premature rupture of the membranes after treatment were also reported [17]. Another trial noted two cases of premature deliveries and one case of bleeding at week 37 after ECV due to excessive pressure on the rear of the placenta [18]. The third trial recorded two cases of premature deliveries and three cases of prelabour rupture of the membranes [19].

## 4. Discussion

In this systematic review and meta-analysis, moxibustion at point *Zhiyin* (BL67) is found to be an effective intervention for correcting non-vertex presentation. With respect to safety, there was no significant difference between moxibustion and control group with outcomes of the use of oxytocin, Apgar scores less than 7 at 5 minutes, cesarean section, preterm delivery, premature rupture of membranes, intrauterine fetal death, placental abruption, and cord blood pH less than 7.1. In case of the use of oxytocin, moxibustion resulted in decreased use of it.

Previous reviews did not include all relevant trials [23–26]. For example, all four reviews failed to include the study of Yang and colleagues [16]. Although the newest Cochrane review (from Coyle and colleagues in January 2012, updated to August 2011) was published within the last two years [26], two high-quality RCTs from Do and colleagues in 2011 and Vas and colleagues in 2013 were not included [19, 21]. Moreover, all these reviews included interventions other than moxibustion. For instance, all four studies included trials, which combined with acupuncture therapy [23–26] or even laser intervention [23]. As we know, moxibustion, acupuncture, and lasers are different interventions. Thus, it is difficult to determine what kind of intervention really works for the correction of non-vertex presentation. 

We made an effort to identify all relevant trials and included high-quality RCTs. Although one study included in this analysis was of lower quality and resulted in high heterogeneity [22], the subgroup analysis that excluded it still showed a favorable effect of moxibustion for the correction of non-vertex presentation. Our study aims to evaluate the clinical efficacy and safety of moxibustion intervention for the correction of non-vertex presentation, so we only included trials comparing moxibustion with non-moxibustion therapy in participants with non-vertex presentation.

Our review has several limitations. Although great efforts were made to retrieve all trials on the subject, there may be still the possibility of missing studies. In addition, some incomplete information may affect the quality and validity of the results. Finally, a large degree of variability of frequency and duration from three times weekly to once or twice daily might be the possible source of bias. 

## 5. Conclusion

The results of our systematic review and meta-analysis showed a positive effect of moxibustion on the correction of non-vertex presentation. In addition, moxibustion might reduce the need for oxytocin. More rigorous high-quality RCTs are still needed to evaluate the efficacy as well as safety of moxibustion for the correction of non-vertex presentation in the future.

## Figures and Tables

**Table 1:** Main characteristics of included RCTs.

Study	Study design	Patient population	Treatment group	Control group	Outcome measures
Yang et al. [16]	Parallel 2-arm	296 participants	Moxibustion at bilateral BL67; twice daily, 30 min each time, 15 min each side; 7 d course (*n* = 147)	Knee-chest therapy; twice daily, 15 min each time (*n* = 149)	NCPCT

Cardini and Weixin [17]	Parallel 2-arm	260 participants	Moxibustion at bilateral BL67; first 87 subjects once daily for 1 week, next 43 women twice daily for 7 d; 30 min each time, 15 min each side (*n* = 130)	Observation; once or twice daily for 30 min each time, 15 min each side (*n* = 130)	(i) NCPDE (ii) NCPCT (iii) CS (iv) UO (v) AS (vi) PD (vii) PRM (viii) IFD

Cardini et al. [18]	Parallel 2-arm	123 participants	Moxibustion at bilateral BL67; twice daily, 30 min each time, 15 min each side for 1 or 2 wk (*n* = 65)	Observation (*n* = 58)	(i) NCPCT (ii) PRM (iii) PA

Do et al. [19]	Parallel 2-arm	20 participants	Moxibustion at bilateral BL67; twice daily, 20 min each time, 10 min each side for 10 d (*n* = 10)	Usual antenatal care for 10 d (*n* = 10)	(i) NCPDE (ii) CS (iii) AS (iv) PD (v) PRM

Guittier et al. [20]	Parallel 2-arm	212 participants	Moxibustion at bilateral BL67; three times weekly; 20 min each time, 10 min each side for 2 wk (*n* = 106)	Expectant management care (*n* = 106)	(i) NCPDE (ii) CS (iii) AS (iv) CBPH

Vas et al. [21]	Parallel 3-arm	270 participants	Moxibustion at BL67; 20 min each time, 2 wk (*n* = 136)	Knee-chest therapy; 20 min each time, 2 wk (*n* = 134)	(i) NCPDE (ii) CS (iii) PD

Yang [22]	Parallel 2-arm	206 participants	Moxibustion at bilateral BL67 + knee-chest therapy; 15–20 min, twice daily, 7 d course for 1 wk (*n* = 103)	Knee-chest therapy, 15–20 min each time, twice daily, 7 d course for 1 wk (*n* = 103)	NCPCT

d: day, wk: week, NCPDE: number of cephalic presentations at delivery (excluding external cephalic version), NCPCT: number of cephalic presentations after cessation of treatment, CS: cesarean section, UO: use of oxytocin, AS: Apgar scores <7 at 5 min, PD: preterm delivery, PA: placental abruption, PRM: premature rupture of membranes, IFD: intrauterine fetal death, CBPH: cord blood pH less than 7.1.

**Table 2:** Additional details of the included RCTs.

Study	Location (country)	Age (mean or range)	Duration	Gestational week	Inclusion	Exclusion
Yang et al. [16]	China	20–36 y	1-2 wk	30–36 wk	Meet the diagnostic criteria, 30 to 34 wk, informed consent, and voluntary acceptance of the experiment	Complicated with pregnancy-induced hypertension, gestational diabetes, merging genital tumor, contracted pelvis, polyhydramnios or oligohydramnios, cord around neck, and fetal biparietal diameter >8 cm, before placenta attach to uterine wall

Cardini and Weixin [17]	China	T: 25.5 ± 2.5 y C: 25.2 ± 3.0 y	1 wk	33 wk	Normal fetal biometry (biparietal and abdominal circumference between percentiles 10 and 90)	Pelvic anomalies, previous uterine surgery, pregnancy-related illness, fetal malformation, twin pregnancy, fibroma > 4 cm, uterine malformation, risk of premature delivery (hypercontractility, Bishop 4 or greater), and tocolysis during pregnancy

Cardini et al. [18]	Italy	T: 31 y C: 26.2 y	1-2 wk	32-33 wk plus 3 d	Normal fetal biometry	Nonacceptance of randomization, pelvic anomalies, previous uterine surgery, fetal malformation, uterine malformation, fibroma > 4 cm, twin pregnancy, previous or current tocolysis, and other pregnancy-related complications

Do et al. [19]	Australia	T: 30.36 ± 3.13 y C: 24.60 ± 5.23 y	10 d	34–36.5 wk	Women were aged greater than 18 years, at 34–36.5 wk of gestation with a singleton breech presentation (confirmed by ultrasound), and normal fetal biometry	Twin pregnancy, risk of premature birth, heart or kidney diseases affecting the mother, placenta previa, history of antepartum haemorrhage, intrauterine growth restriction, hypertensive disease, isoimmunisation, previous uterine operations, uterine anomaly, prelabour rupture of the membranes, multiple pregnancy, fetal congenital abnormality, contraindication to vaginal delivery, and fetal death in utero

Guittier et al. [20]	Switzerland	T: 32.0 ± 4.3 y C: 32.0 ± 4.2 y	2 wk	T: 35 ± 0.8 wk C: 34.8 ± 0.7 wk	Single fetus in breech presentation between 34 and 36 wk of gestation	Uterine malformation, placenta praevia, and transverse lie

Vas et al. [21]	Spain	T: 22.6–39.0 y C: 24.0–38.3 y S: 24.4–38.0 y	2 wk	33–35 wk	Diagnosed by physical examination and ultrasound; at least 18 years; 33–35 wk of gestation (confirmed by ultrasound); normal fetal biometry and no prior treatment with moxibustion to achieve version of the fetus	Multiple pregnancy, bone pelvic defects, previous uterine surgery, fetal malformation or chromosomal disorder, uterine malformations, risk of preterm birth (preterm uterine contractions and/or initial dilatation or shortening of the cervix with a score of 4 on the Bishop scale), uterine fibroids >4 cm, tocolytic therapy, and maternal heart or kidney disease

Yang [22]	China	T: 26–28 y C: 25–27 y	7 d	28–32 wk	Not stated	Not stated

T: treatment group, C: control group, S: sham group, y: year, wk: week, d: day.

**Table 3:** Risk of bias of included RCTs.

Study	Random sequence generation	Allocation concealment	Blinding of participants and personnel	Blinding of outcome assessment	Incomplete outcome data	Selective reporting	Other bias
Yang et al. [16]	Computer generated	Sealed envelopes	Not stated	Analyst was blinded	7 subjects from treatment group and 10 subjects from control group withdrew from the trial	SPUU	IID

Cardini and Weixin [17]	Computer generated	Sealed envelopes	Neither participants nor practitioner was blinded	Not stated	Complete followup of all subjects	SPUP	NIR; SAF

Cardini et al. [18]	Computer generated	Sealed envelopes	Neither participants nor practitioner was blinded	Assessor was blinded	1 subject in control group was lost to followup; 14 subjects in intervention group discontinued treatment	SPUP	TIIA

Do et al. [19]	Computer generated	Sealed envelopes	Not stated	Analyst was blinded	1 subject in control group was lost to followup, but less than 10%	Study protocol available	NIR; SAF

Guittier et al. [20]	Computer generated	Sealed envelopes	Not stated	Not stated	Complete followup of all subjects	SPUP	NIR; SAF

Vas et al. [21]	Computer generated	Sealed envelopes	Participants in true and sham moxibustion groups were blinded	Analyst was blinded	Complete followup of all subjects	Study protocol available	NIR; SAF

Yang [22]	Table of random numbers	Not stated	Not stated	Not stated	Followup of all subjects was not reported	SPUU	IID

SPUU: study protocol unavailable; unable to determine whether all outcomes were prespecified, SPUP: study protocol unavailable, but published report includes all expected outcomes, IID: insufficient information to determine whether the other bias is present, NIR: no imbalances at randomization, SAF: study appears free of other sources of bias, TIIA: trial was interrupted when interim analysis revealed poor compliance and a high number of treatment interruptions.
